# Barriers to adopting digital contact tracing for COVID‐19: Experiences in New Zealand

**DOI:** 10.1111/hex.14013

**Published:** 2024-03-16

**Authors:** Phoebe Elers, Tepora Emery, Sarah Derrett, Tim Chambers

**Affiliations:** ^1^ Department of Public Health University of Otago Wellington New Zealand; ^2^ Research Office Toi Ohomai NZIST Rotorua New Zealand; ^3^ Ngāi Tahu Māori Health Research Unit, Division of Health Sciences University of Otago Dunedin New Zealand

**Keywords:** communication inequality, COVID‐19, digital contact tracing, NZ COVID Tracer app

## Abstract

**Background:**

Digital contact tracing (DCT) was a central component of the global response to containing COVID‐19. Research has raised concerns that DCT could exacerbate inequities, yet the experiences of diverse communities at greater risk from COVID‐19 are typically underrepresented.

**Methods:**

The present study aimed to understand the perceived barriers to the adoption of the app amongst Māori, Pasifika, and disabled people. Focus groups and interviews were undertaken with Māori, Pasifika, and disability sector stakeholders and community participants.

**Results:**

Participants (*n* = 34) generally expressed willingness to utilise DCT and support its adoption within the communities. Simultaneously, participants revealed how the app could marginalise community members who struggled with the usability and those distrusting of the government's COVID‐19 interventions.

**Conclusions:**

The findings highlight how addressing communication inequality can assist in the development of contact‐tracing responses that are both effective and equitable. The study provides insights about the role of information and communication technologies as health resources.

**Patient or Public Contribution:**

Consulting with members of the target communities was central throughout the present study, including recommendations for potential participants, participation in interviews and sharing early findings for feedback. This study reports on focus groups and interviews with individuals from Māori and disability sectors.

## 1 INTRODUCTION

A central component of the global response to containing COVID‐19 was *digital contact tracing* (*DCT*)—the use of information and communication technologies (ICTs) to track, trace or inform contacts of infectious cases. DCT is frequently employed through an ‘app’ that individuals download onto their smartphones.^1^ Research examining public perspectives of DCT has identified barriers to adoption, including low access to ICTs, apprehensions regarding cybersecurity and privacy and a lack of trust in the government,^2^ raising concerns that DCT could have a lower uptake amongst some groups who are at greater risk of more severe illness from COVID‐19.^1^ However, the perspectives and experiences of these higher‐risk groups of DCT have been underrepresented in qualitative research (for a notable exception, see O'Donnell et al.^3^). This is problematic, as it underrepresents these communities' information needs, the systemic and structural barriers to adoption and how current systems could be reconfigured to better serve communities.

The present study centres the adoption barriers identified by Māori (the Indigenous population in New Zealand [NZ]), Pasifika (referring to the Indigenous populations of the Pacific Islands) and disability sector stakeholders and community participants regarding the New Zealand COVID Tracer App (NZCTA)—the DCT app utilised in NZ (also known as Aotearoa or Aotearoa me Te Waipounamu). While highly heterogenous, Māori, Pasifika and disabled people are, at a population level, statistically at greater risk of adverse COVID‐19 outcomes^4^, ^5^ and are also more likely to have lower overall levels of access to the internet,^6^ but are underrepresented in academic research on the NZCTA. By highlighting these statistics and underrepresentation, we emphasise the need to foreground voices from, or in close connection with, these communities in evaluating the NZCTA.

### 1.1 Background to the study

DCT apps, by nature, relied on high public adoption to be useful in containing COVID‐19.^1^ The effectiveness of DCT is unproven, as there are few published quantitative studies in real‐world outbreak settings.^1^ DCT apps can vary significantly; the NZCTA used a decentralised architecture that stored data on personal devices and is thus, more privacy preserving than apps using a central server. The app employed location tracking through scanning posters with QR codes. From August 2021 to March 2022, record‐keeping was mandatory upon the entry of premises through QR scanning or manual recording. In December 2020, a Bluetooth proximity detection option was added. The COVID‐19 Public Health Response Act 2020^7^ specified the circumstances under which the information from the NZCTA could be used or disclosed. The NZCTA was released in May 2020, the requirement for QR code scanning was lifted in March 2022, and it was subsequently removed from app stores in 2023.

Research has contributed valuable insights into the acceptability and barriers associated with the NZCTA.^8^, ^9^, ^10^ Tretiakov and Hunter^10^ observed that privacy considerations had a limited impact on app adoption. A separate study conducted focusing on contact tracing professionals' perspectives of DCT in NZ highlighted concerns related to equity, placing undue burden on the public, and increasing the workload for the existing contact tracing system.^11^ Our study focuses on Māori, Pasifika, and disabled sector stakeholders and community participants because these groups statistically have a high risk in relation to COVID‐19 infection,^4^, ^5^ lower overall levels of access to the internet,^6^ and are underrepresented in DCT scholarship. Māori, comprise 16.5% of the national population.^12^ The Pae Ora (Healthy Futures) Act^13^ specifies that the health sector must adhere to the principles of equity, ensuring that Māori and other population groups have access to services proportionate to their needs, receive equitable levels of service and achieve equitable health outcomes.

The colonisation of NZ led to significant land loss, infectious disease epidemics and cultural assimilation policies, which contributed to an urban drift of many Māori from the 1950s.^14^ This was followed by an increase in migration to NZ from Pacific nations.^15^ Pasifika peoples currently comprise 8.1% of the national population.^12^ Approximately 24% of the national population identifies as disabled.^16^ Disabled people (the term ‘disabled people’ aligns with the Office for Disability Issues^17^ following guidance from the New Zealand Disability Strategy Revision Reference Group) continue to experience lower incomes and access to education in NZ,^18^ and are often excluded from decision‐making processes related to laws and health interventions impacting their lives.^19^, ^20^ Against this backdrop, we explored: *What are the perceived barriers to adoption of the NZCTA amongst Māori, Pasifika, and disabled people?*

## 2 MATERIALS AND METHODS

All study procedures were approved by the University of Otago Human Ethics Committee (HE20/010). The focus groups and interviews were conducted between February and July 2023. Being an interdisciplinary team that included Dr Tepora Emery, who is a kaupapa Māori researcher,^21^ but is predominantly non‐Māori and nondisabled (although with experience working with the communities of interest), it was crucial to examine our positionality and our own inherent biases and predispositions throughout the research processes. While our research is not underpinned by a co‐design, Pacific or kaupapa Māori methodology, being part of a broader evaluation that examined the perspectives of multiple participant groups, our study processes were informed by Māori, Pacific, and disability research guidelines.^22^, ^23^, ^24^ This meant that taking time to consult with community members was vital throughout the research and we engaged with community representatives in the planning phase regarding the participant recruitment, study procedures and interview questions (comprising six Māori leaders and two disability providers (three face‐to‐face, two via Zoom and two via phone). The interview guide remained largely unchanged following these interactions, but it played a key role in shaping our recruitment strategy. We were unable to engage with Pacific representatives in the planning phase but met with Pacific representatives later in the project who recommended organizations to approach for recruitment), offered opportunities for participant feedback, and shared preliminary project findings with consenting participants for their input.

### 2.1 Methodological approach

Our research was underpinned by Bijker et al.'s^25^ social construction of technology (SCOT) theory, which posits that emerging technologies evolve and gain new meanings through social interactions amongst relevant social groups. Different social groups can adopt varied perspectives and meanings of a single technological artefact^26^ and SCOT researcher focus on the social contexts of technology development and how users adapt innovations to their needs.^26^ This involves reconstructing technology interpretations through the eyes of relevant social groups.^25^ We have also drawn on the work of Klein and Kleinman^27^ by considering dynamics of structural power disparities across social groups that can act as both facilitators and impediments in technological adoption and design.

### 2.2 Participant recruitment

The identification of relevant social groups relies on seeking out those with commonalities in relation to the technological artefact of interest, aligning with purposive and snowball sampling methods.^28^ Our participants were initially identified through a mapping process, with input from six Māori leaders and two disability providers, to identify key disability sector stakeholders and community participants to approach for recruitment. Emails were sent to individuals identified providing information about the study, inviting them to participate in focus groups (alongside their respective groups) and asking for their recommendations of other contacts who could be invited.

### 2.3 Interview and focus group conduction and analysis

The first author conducted all interviews and focus groups, with five out of the six focus groups additionally facilitated by a second researcher chosen for their expertise in engaging with the community participants represented. We endeavoured to accommodate participants' needs whenever possible. For those unable to join during the scheduled focus group times, alternative arrangements were organised, allowing them to participate either in another focus group or an individual interview. Disability sector participants were consulted to ascertain if any accommodations were necessary to ensure their full engagement. The research used a blend of online and in‐person interactions, with interviews and five focus groups being conducted and video‐recorded via Zoom, and one focus group being conducted and audio‐recorded in person to respect participant preferences. The open‐ended focus group and interview questions explored various aspects related to the participants' experiences and perceptions regarding the NZCTA during different stages of the COVID‐19 pandemic. Each of these questions was followed by a probe that inquired about community‐level perceptions or experiences related to the topic. Analyses did not focus on distinctions between various forms of disability. All focus groups and interviews were conducted in English.

Transcriptions of interviews and focus group sessions were completed verbatim and then anonymised; participants were assigned pseudonyms. Anonymised transcripts were subsequently analysed using Braun and Clarke's^29^ reflexive thematic analysis on NVivo. Reflexive thematic analysis is compatible with SCOT, being a theoretically flexible interpretative approach to qualitative data analysis that involves identifying initial codes from participant responses and organising these codes into potential themes that are later refined and named. The reflexive approach emphasises the researchers' active involvement in the process of knowledge generation.^29^ Within this framework, codes are considered as the researchers’ interpretations of discernible patterns of meaning within the data set. Reflexive thematic analysis is thus, a result of the researchers' reflective and interpretive examination of the data, carried out at the juncture of the data set itself, the study's theoretical underpinnings, and the researcher's analytical skills and resources.^29^ One qualitative researcher led the analysis and a kaupapa Māori researcher cross‐checked a coded transcript of a focus group with Māori sector participants and another team member cross‐checked two different coded transcripts. All researchers met to discuss the findings, including themes. A summary of the findings was shared with participants and members of the Ministry of Health, allowing them time to provide feedback.

## 3 RESULTS

Six focus groups (with a combined total of 30 participants) and four individual interviews were undertaken in 2023 lasting 60–90 min. Participants comprised Māori (*n* = 10), Pasifika (*n* = 15) and disability (*n* = 11) sector stakeholders and community participants, including two participants who identified with two groups. Participants had a range of demographic characteristics (presented in Table 1) and represented various community, health and disability organisations (six Māori organisations, four Pasifika organisations and eight disability organisations), including two Māori and two Pasifika churches. Not all disability sector participants identified as having a disability or lived experience of impairment (and for the eight participants who did, this did not impede their ability to provide informed consent). Participants who identified as disabled included people living with intellectual, visual and/or physical impairment. One Māori sector participant did not identify as Māori, but who worked for a Māori organisation that served Māori. All Pasifika sector participants identified as Pasifika.

**Table 1:** Participant demographics.

Demographic	Number of participants (total = 34)	Proportion of total (%)
Age		
18–34	2	6
35–54	16	47
55+	10	29
This data is missing	6	18
Gender		
Male	16	47
Female	18	53
Ethnicity^a^		
Māori	9	27
Pasifika	16	47
NZ European	9	27
Other	1	3
Sector group^a^		
Māori	10	29
Pasifika	15	44
Disability	11	31

^a^ Participants could identify as multiple ethnicities and represent more than one sector group, so figures exceed the number of participants in these categories. Participants who identified as Pasifika included Samoan, Tongan, Fijian, Niuean and Cook Island ethnicities.

This study if focused on the barriers to adoption of the NZCTA identified by Māori, Pasifika, and disability sector participants. However, it is crucial to acknowledge that most participants embraced and used the app. Participants deemed the NZCTA to be ‘necessary’ [Dilara‐Disability‐Sector] with Māori and Pasifika sector participants emphasising a responsibility in using the app for the ‘safety of your family’ [Jacob‐Māori‐Sector] and society at large. Participants described supporting others to use the app, as ‘When you have kuia [elderly relatives] out there, their COVID tracers were their mokopuna [grandchildren], their children. They were the COVID tracer apps for them’ [Emma‐Māori‐Sector].

Simultaneously, our analysis identified two themes that show how the NZCTA could marginalise community members who did not use the app or struggled to use it. In the first theme, we found *exclusion* was intwined with the inaccessibility of both the NZCTA and its promotional material, as particularly emphasised by disability sector participants. Yet for many Māori and Pasifika sector participants, the NZCTA was also situated against a broader context that is tied to the disengagement of the COVID‐19 response. The second theme, *Distrust*, is centred on the interplay between distrust and disinformation hindering the app's adoption, set against a colonial backdrop for some Māori sector participants. These themes show how addressing communication inequality stands at the core of creating equitable strategies for future pandemic responses.

### 3.1 Theme 1: Exclusion

#### 3.1.1 Inaccessibility

Participants reported that the NZCTA exacerbated the existing digital divide amongst individuals and communities. While many participants were able to adopt the NZCTA into their daily lives, they pointed out how others, notably elderly, lower socioeconomic groups, and some disabled people, encountered barriers in using or accessing smartphones. Participants stated: ‘Some people didn't have phones’ [Adelyn‐Pasifika‐Sector] and ‘They only knew how to text and pick up a phone call … I thought [the app] was a bit stressful for some of our *kaumātua* [elders]’ [Marama‐Māori‐Sector]. Usability issues were most strongly emphasised by disability sector participants with the app being unable to accommodate various disabilities; for example, for those with low vision or mobility impairments when wanting to scan the QR posters, and for those with learning impairments.

Disability sector participants pointed to how accessibility could have been improved by incorporating smartphone accessibility guidelines, discussing the need for colour contrast, haptic feedback and read‐aloud functions. A small number of participants felt that Bluetooth proximity detection could assist in addressing certain accessibility issues. However, a stronger emphasis was on the importance of offering multiple contact tracing options as: ‘Not all the options fit all the people’ [Beth‐Disability‐Sector] and ‘More needs to be done to put these tools in the hands of people… but having other options as well would address the fact that nobody's left behind’ [Iris‐Disability‐Sector].

There was a consequent emotional toll for those who struggled to use the app when record‐keeping was mandated for entry to premises. Consider Dilara's [Disability‐Sector] experience:

I was really concerned… I always tell my, my husband, ‘what should I do if I cannot scan?’ [from my wheelchair] … When you enter the business … some of them are very, very carefully look at you to see if you scan your phone and if you didn't… They told you that you should… They ask you, they *force you*… It was embarrassing. [Italicised font said with emphasis]

Dilara's description highlights the social impact of digital exclusion from the design of the NZCTA that did not cater to her disability. Although manual recording (writing contact information on designated forms) was an alternative option to QR scanning when entering premises, disability sector participants described how manual recording could also be difficult for some disabled people and could breach people's confidentiality and privacy as their personal contact information was publicly viewable, making them ‘vulnerable’ [Iris].

Issues in locating or reaching QR posters were discussed by many disability sector participants, which could lead to frustration and concerns about inconveniencing others when disabled people blocked doorways or required assistance. As Greg [Disability‐Sector], who is blind, recalled: ‘For a blind person, how do you find the … [QR] poster in the first place?… I certainly have handed my phone to my partner or my child saying, ‘I can't find this damn QR code’. Disability sector participants expressed that relying on others to assist with DCT ‘takes away people's independence’ [Caroline], which ‘impacts on people's dignity’ [Dilara].

#### 3.1.2 Information dissemination

The exclusion was also perpetuated through inequitable information dissemination about the NZCTA and the broader COVID‐19 response. Although some participants felt that aspects of the NZCTA's promotion were effective, all participant groups recommended further promotional efforts for future responses, particularly about how to use DCT tools. Māori and Pasifika sector participants suggested that messaging should be culturally appropriate and delivered in different languages (including for the app's interface) by: ‘…community leaders … who are respected… For our Tongan community there are very clear, you know, community structures…’ [Anitelu‐Pasifika‐Sector].

Disability sector participants, again, revealed the social impact of this exclusion for disabled people, as ‘Not everybody has a great sense of a support network around them to inform them, to keep them up to date, so they were left alone, not knowing’ [Caroline]. Despite disability sector participants discussing how access to health information in disability‐accessible formats is required in the United Nations Convention on the Rights of Persons with Disabilities,^30^ it was clear that this was not materialised during the pandemic and ‘The challenge was getting some of that information out to people’ [Iris]. A disability sector participant who assisted in the dissemination of information in accessible formats explained that it was difficult because ‘Information kept changing… You needed stable information to do the accessible formats, of which there's um more than easy read. So, sign language, Braille… large font’. [Caroline]. Another participant responded:

I know we can't embargo, but I almost feel that that's what should happen… It speaks about that umm systemic discrimination of ableism around that people do not consider everybody up front… It always feels that disabled people are tagged on afterwards, like ‘we'll get it out, and then we'll get to them’, and that shouldn't be the way it's done. [Elena‐Disability‐Sector]

In the above excerpt, Elana expresses frustration in the delay in disseminating information to disabled people. Her revelation that it ‘always feels’ this way speaks to a history of the exclusion of disabled people. Iris expressed a similar frustration: ‘Disabled people were, were at the bottom of the cliff… Disabled people are part of this country's population, and their needs are [as] important as everybody else's’. Disability sector participants called for a shift in approach, advocating for disabled people to be included from the offset in planning and decision‐making.

Across the participant groups, participants emphasised the importance of consulting and involving communities in the development of DCT, including amongst those who were satisfied with its design. While some participants recalled positive experiences of Māori and Pasifika organisations collaborating in COVID‐19 efforts, many recommended further engagements with communities in future DCT responses. Māori sector participants recommended: ‘Partnership with our iwi, with our Māori health providers, that they be at the forefront of the conversation, that they be part of the solution’ [Scott], ‘te ao Māori [Māori worldview] is a part of the solution. Get us to a part of the design and ugh get us to the table earlier’ [William], and ‘by Māori for Māori solutions… They are the most effective’ [Max].

### 3.2 Theme 2: Distrust

#### 3.2.1 Disinformation and distrust

While participants generally portrayed a willingness to adopt the NZCTA, Māori and Pasifika sector participants revealed a specific community subset where distrust interworked with disinformation and impeded their adoption. Participants stated: ‘Lots of sources of information … were not reliable’ [Kalauni‐Pasifika‐Sector], ‘Distrust I guess… That making a difference for them’. [Kaia‐Māori‐Sector], and:

Some of our community don't have permanent residency … [They] weren't comfortable in disclosing or downloading anything like that [app] as much as they wanted to, because they're scared for their immigration status… If you have a family member or you live with someone who doesn't have permanent residency there's that fear that none of them can use it, because then they'll all be traced, and it will come back to them… [Mele‐Pasifika‐Sector]

Other participants in the focus group signalled agreement with Mele's description. NZ has a history of deporting Pasifika peoples who overstay their residency, and news articles reported that this continued in 2020, even in regions where healthcare facilities were overwhelmed by patients infected with COVID‐19.^31^ The fear Mele describes is not solely about the NZCTA or pandemic response but encompasses the wider social, legal, and familial ramifications that are intertwined with immigration. The notion of trust (or a lack thereof) is central—the reluctance described extends beyond the app, manifesting deeper distrust in the systems that govern it.

Moreover, some participants felt that disinformation worsened through the pandemic, whereby the: ‘…deeper we got into the pandemic … misinformation and distrust got worse’ [Sophia‐Māori‐Sector]. This intertied with the feeling of ‘…anti‐government … anti‐this anti‐that’ [Scott‐Māori‐Sector], and ‘anti‐anything to do with COVID’ [Kaia‐Māori‐Sector], which created: ‘…a wide division… It wasn't against the application, specifically, it was against … all what was happening at the time’ [Marama‐Māori‐Sector]. These excerpts reveal how escalating misinformation and intertwining distrust during the pandemic eroded faith in specific sources of information, including of the NZCTA, and contributed to an environment of social divide within the community.

#### 3.2.2 Colonial backdrop

When asked about the barriers and enablers to the adoption of the NZCTA, Jacob [Māori‐Sector] responded that it was ‘Bad timing off the back of that Oranga Tamariki [Ministry for Children], Royal Commission Inquiry… The taking of the children’. Oranga Tamariki had gained media attention due to the disproportionate up placements of Māori children away from the families and into state ‘care’, while the Royal Commission of Inquiry^32^ reported on the extensive overrepresentation and abuse of Māori and Pasifika within this state ‘care’ system. Jacob's perspective, linking app reluctance to distrust resonated with other Māori sector participants' observations of community members with ‘massive, like understandably, mistrust in the government’ [Sophia].

This context of distrust established through colonisation contributed to an environment for disinformation to be spread. As Max [Māori‐Sector] explained: ‘A lot of these [disinformation] groups were able to build on … that misinformation, because of the, the decades of mistrust between government and, and Māori communities’, highlighting how historical (and contemporary) grievances can impact challenges in information dissemination. Max further stated:

It builds on the distrust… Government needs to take responsibility… They've just done so many things over decades. They have just built that mistrust between Māori and the government…. It's like ‘We're just gonna do shitty things to you, ugh, but now we need you to trust us’… There's still a lot of … healing and, and restoration and trust building that needs to happen particularly in in in Māori communities. The mistrust that they have for government *is absolutely justified*. Absolutely justified. [Italicised font was said with emphasis]

Max's excerpt underscores the far‐reaching impact of governmental actions and the substantial efforts needed to rebuild trust with some Māori communities.

## 4 DISCUSSION

Our study centres on the barriers to adopting the NZCTA expressed by Māori, Pasifika, and disability sector stakeholders and community participants—groups that have previously been underrepresented in NZCTA research. While most participants embraced and utilised the app and supported community members to do so, participants articulated two central concerns of the NZCTA within the broader context of the pandemic. One concern, conveyed by Māori and Pasifika sector participants, was how the app could be framed within narratives of disinformation, grounded in distrust. The other concern focused on the inequitable accessibility of the NZCTA and the resulting exclusion, perpetuated through inequitable information dissemination about the NZCTA.

Research has documented how COVID‐19 advice competed with unfiltered online information sources.^33^ In relaying concerns about how the app could be framed within narratives of disinformation, participants revealed how the context of distrust is tied to Crown structures that have marginalised Māori and Pasifika communities.^32^, ^34^ This contributed to an environment conducive to the dissemination of disinformation and impeding the app's adoption. Previous research examining perspectives of the NZCTA,^8^ and of DCT apps in other settings^2^ have similarly identified a lack of trust as a significant barrier to adoption. For instance, O'Donnell et al.^3^ emphasised the importance of trust in the overall system, not just DCT apps, stating that many individuals expressed concerns about their data potentially being shared for benefits checks or for those in search of asylum. Our research highlights how distrust is deeply embedded in social contexts, entrenched in a colonial history that has systematically marginalised community members.

Other DCT research has overlooked participant (dis)trust, focusing primarily on privacy concerns as isolated barriers to technology adoption and informing recommendations to enhance privacy features of digital tools.^35^ This is exemplified in the more privacy‐preserving decentralised architecture in some DCT apps, including the NZCTA. However, frameworks which do not account for the social contexts in negotiating technology may neglect the role that distrust plays in exacerbating privacy concerns amongst specific population groups. In our findings, privacy was not a central concern for many participants; instead, they emphasised how distrust in the COVID‐19 response amplified privacy apprehensions within their communities. Notably, when discussing distrust and privacy issues, participants did not mention the decentralised nature of the app, indicating the need for heightened promotion efforts. This is significant because it implies that the app's decentralised nature may require a high level of digital literacy for understanding, potentially failing to reassure some community members who are sceptical of the government's COVID‐19 response. This aligns with Ndayishimiye et al.'s^36^ ^,p.281^ recommendation to educate the public about DCT, especially individuals with lower levels of health literacy, emphasising that ‘inclusiveness and equity should be ensured to keep up with the changes that are necessary to provide care for all’.

The second concern regarding the inequitable accessibility of the NZCTA was voiced by all participant groups in the context of smartphone accessibility, but disability sector participants particularly emphasised issues related to usability, echoing other research that has also documented challenges in the accessibility of DCT amongst already marginalised groups.^36^ It is evident from our research that the NZCTA did not accommodate various disabilities. A troubling consequence was an unprecedented level of inequity in access for some disabled people, impacting not only individual premises but every premise across the country during phases of the pandemic response. This pervasive inaccessibility, combined with participants' perceived lack of consultation with disabled people and advocacy organisations in the development of the NZCTA, conflicts with the United Nations Convention on the Rights of Persons With Disabilities^30^ and NZ's disability strategy.^17^ The underutilisation of these critical resources, developed to safeguard the fundamental rights of disabled people, exposes persistent systemic challenges and ableist discrimination. The failure to accommodate and meaningfully consult disabled people in the NZCTA, as perceived by disability sector participants, exacerbates inequity and perpetuates their erasure by denying recognition of their unique experiences, needs and inherent dignity. It is another example of the exclusion of disabled people from decision‐making processes that directly impact their lives.^19^

The need for engagement and consultation with the communities in developing DCT responses emerged as an area of convergence across the participant groups. Increasing community engagement was viewed as a means to address the core concerns associated with the NZCTA—distrust and inequitable accessibility—both of which hold the potential to marginalise community members. Our research further speaks for the need for broader systemic change and transformation; it is ineffectual, for example, to seek Pasifika groups' input on DCT privacy concerns while simultaneously deporting individuals who have overstayed their visas.^31^ Our research suggests that systemic injustices can erode trust within communities, which can lead to inequitable access to DCT tools. This, in turn, perpetuates the problem further, as inadequate engagement with these groups deepens the cycle of distrust and exclusion.

In 2020, a staff member at the National Investigation and Tracing Centre questioned: ‘If we're not harnessing technology to promote equitable outcomes, is its use warranted at all?’.^11^ ^,p.5^ This question posed following the release of the NZCTA highlights concerns about inequity, prompting further scrutiny of the app's mandating. Despite disability sector participants suggesting that equity could be improved through more accessible ICTs, a single technological tool cannot cater to the diversity of humanity. Root issues of distrust raised by Māori and Pacific sector participants cannot solely be resolved through technology. A critical concern in the staff member's question is its timeliness; it should be addressed as a starting point, not an end point, captured in the disability rights catchphrase ‘nothing about us without us’. Placing equity at the forefront is crucial for building more effective pandemic responses and DCT tools that better serve the communities.

## 5 STUDY LIMITATIONS

In keeping with the qualitative nature of our research, our findings may not be transferable to other contexts and settings. Our study did not explicitly explore the intersectional influences of various identities, nor extensively investigate specific technological barriers faced by different disability‐specific communities. The focus groups and interviews were all undertaken in English. It was beyond the scope of our research team and the timeline of the study to arrange and undertake interviews in different languages but such qualitative research could generate a wide range of important perspectives. That said, strengths of our qualitative methodology include the focus on perspectives of people from communities known to be at increased risk of adverse COVID‐19 outcomes—groups which were, and continue to be, overlooked in policy‐making and programme implementation. Future quantitative research could usefully explore with larger numbers of participants the potential of strategies identified in our study. For example, ensuring the best options are identified to improve app accessibility across a range of disability, Māori and Pacific communities and to genuinely engage with these communities to build trust. Such work should commence now to improve DCT strategies available and ready to implement rapidly in advance of future, sadly inevitable, pandemics.

## 6 CONCLUSIONS

The study provides insights into the barriers to adopting the New Zealand NZCTA amongst Māori, Pasifika, and disabled communities. While many participants embraced the app, distrust grounded in marginalisation could frame the NZCTA within narratives of disinformation, potentially hindering its adoption. Participants also revealed the inequitable accessibility of the NZCTA, particularly disadvantaging the elderly and people with disabilities. These findings highlight the need to prioritise equity in pandemic responses and to engage with communities now in developing health and DCT tools.

## AUTHOR CONTRIBUTIONS

**Phoebe Elers**: Writing—original draft; investigation; methodology; conceptualisation; writing—review and editing. **Tepora Emery**: Conceptualisation; methodology; investigation; writing—original draft; writing—review and editing. **Sarah Derrett**: Conceptualisation; methodology; investigation; writing—original draft; writing—review and editing. **Tim Chambers**: Conceptualisation; methodology; investigation; funding acquisition; writing—original draft; writing—review and editing.

## CONFLICT OF INTEREST STATEMENT

The authors declare no conflict of interest.

## ETHICS STATEMENT

All study procedures were approved by the University of Otago Human Ethics Committee (HE20/010). All participants completed informed consent processes.

## Data Availability

The data from our qualitative study cannot be made publicly available in compliance with our university's ethical data storage requirements, as releasing the recordings or transcripts could potentially compromise the anonymity of our study participants
